# The Value of Compression of the Aorta in the Treatment of Post-Partum Hemorrhage

**Published:** 1907-12

**Authors:** F. Percy Elliott

**Affiliations:** Medical Officer to the Walthamstow Public Dispensary.


### THE VALUE OF COMPRESSION OF THE AORTA IN THE TREATMENT OF POST-PARTUM HEMORRHAGE.

(*Continued.*)

BY

F. PERCY ELLIOTT, M.B.,

*Medical Officer to the Walthamstow Public Dispensary.*

SINCE the publication in the June number of this *Journal*, Vol. XXV., p. 121, of a case illustrating the value of compression of the aorta in the treatment of post-partum hemorrhage, I have received many communications bearing on different aspects of the question. The treatment of post-partum hemorrhage in general practice, owing to the conditions which prevail, will always be more curative than preventative, and the circumstances at the early part of this case illustrate a state of affairs bearing out this point, and common enough in general practice. The patient was seen by my assistant when the os admitted the tip of one finger, and the labour pains feeble and irregular. Bleeding had ceased, and had not been sufficient to produce any ill-effect on the general condition of the patient, and an anæsthetic was necessary. Owing to all these circumstances, delay occurred in immediately performing version. The manner of delivery did not predispose to post-partum hemorrhage. After version was performed and a leg brought through the os, beyond keeping the child on the lower uterine segment between the pains, no interference took place until the shoulders appeared, when delivery was completed in the usual way. The comparatively rapid delivery was due to the onset of strong and frequent labour pains after a leg was brought through the os, an easily dilatable os, and to the good down-bearing efforts of the patient. The patient was not kept deeply under the anæsthetic except during the time that version was being performed.

The reasons for recording the case were to show that manual compression of the aorta, despite the assertions to the contrary made in the recent debate on the subject, (1) is a practical measure ; (2) that under certain conditions it is the only method that can be relied on for rescuing the patient from death ; (3) and that it is not necessarily attended by harm when it is employed, (*a*) before delivery of the placenta for arresting hemorrhage between the second and third stages of labour, or (*b*) when it is employed after delivery of the placenta. The case recorded serves well to illustrate these points.

**Compression of the aorta as the primary part of the treatment of the hemorrhage occurring between the second and third stages of labour.** After a leg was brought through the os, no further bleeding occurred until the head was delivered. The hemorrhage at this stage was obviously due to partial separation of the placenta, an incident usual when the placenta is implanted on the lower segment of the uterus. The usual proceeding in serious hemorrhage is straightway to resort to manual separation and removal of the placenta. The cause of hemorrage, it is taught, must be removed at once. No rest is given the uterus, no opportunity afforded for natural separation of the placenta to take place; the hand is introduced into the uterus immediately, the placenta hurriedly removed. Nothing is done to obviate the necessity for this. Further, while manual removal of the placenta is being carried out, nothing is done to arrest hemorrhage; it is taken for granted that the patient will be none the worse for the additional loss of blood, and that as soon as the cause of the hemorrhage is removed the uterine forces will immediately become adequate, and remain so.

The conditions in this case demonstrate well the advantages of compression of the aorta as the primary part of the treatment of hemorrhage occurring in the interval between the second and third stages of labour.

1. It gave rest to the uterus, a desirable thing in all cases after the child is born, and especially when severe loss of blood has occurred. In the case under consideration, although there were no signs of inertia up to the time of delivery of the head, with the sharp bleeding which followed immediately, inertia appeared.

2. In addition to affording rest, compression of the aorta prevented the further exhaustion of the uterus, which would have occurred from the continued loss of blood which is inevitable until the uterus is emptied.

3. In virtue of this rest and recuperation, opportunity was given for natural separation of the placenta to take place. Complete separation of the placenta occurred within half an hour after the birth of the child, as was proved by exploration of the cavity of the uterus. Moderate pressure on the uterus was all that was required for the complete extrusion of the placenta.

4. Risk of sepsis was minimised. Owing to hurried removal of the placenta in the natural desire to remove it as quickly as possible when hemorrhage has been severe and the life of the patient is at a low ebb, small pieces of placenta are likely to be left adhering to the uterine wall, and to be a source of sepsis subsequently. Scraping the placental site to remove such pieces of placenta is also not infrequently rendered necessary by hurried removal of the placenta. These sources of danger become much greater when the placenta is morbidly adherent. Although in this case it was necessary to explore the uterus owing to the raged condition of the lower portion of the placenta, it is not always necessary to do so in cases of early partial separation of the placenta, careful examination of it after delivery alone being often quite sufficient to obviate the proceeding. Had it become necessary in this case to remove the placenta ultimately, with the hemorrhage arrested by compression of the aorta, the risks consequent on hurried removal would have been avoided.

5. Danger of subsequent hemorrhage from retained pieces of placenta, the result of hurried removal when bleeding is severe, was also avoided.

6. The danger to life from the additional hemorrhage when the placenta is removed manually and without any steps for the arrest of hemorrhage meanwhile, was also avoided. This danger is a very real one when the patient has been already seriously drained of blood, especially when the placenta is morbidly adherent. There was no obvious feature present in this case to show that the removal of the placenta could be accomplished without great loss of blood, and although bleeding might not have been great, it would certainly have added greatly to the peril of the patient. Prompt arrest of hemorrhage was of vital importance, as, in spite of immediate occlusion of the aorta when the hemorrhage occurred, the general condition of the patient at once became very grave.

7. In addition to treating shock by arresting hemorrhage, compression of the aorta also treated shock far more quickly and in a much more effectual manner by suppressing the circulation of blood in the lower extremities. The vital centres were thus kept better flushed with blood.

In view of all these facts, I think that compression of the aorta as the primary part of the treatment of the hemorrhage at this stage was the best step to take in this case. The result fully justified its employment. At the time when its application took place the condition of the patient gave cause for great anxiety, her pulse rising rapidly from 130 to 150 and becoming threadlike, uterine inertia appearing, and signs of general collapse being present. After compression of the aorta, within three hours, the pulse had dropped to 120, the uterus had become firmly contracted, no bleeding occurring after releasing compression, and all signs of collapse had passed off. Compression lasted for about an hour.

**Compression of the aorta as the primary part of the treatment of the secondary post-partum hemorrhage.** The hemorrhage in this case occurred suddenly, after a period of over eight hours' freedom from bleeding. I regret that I omitted in my former paper to mention the circumstances which attended and appeared to determine the onset of secondary post-partum hemorrhage in this case. It was stated that the patient was of an excitable nature, and it was following an outburst of mental and physical excitement that the hemorrhage occurred. The uterus, it will be remembered, had been previously thoroughly explored; no placental remains were present, and the organ had been completely emptied, and good contraction and retraction secured. There was, therefore, no question of retained portions of placenta or chorion. As secondary post-partum hemorrhage is not uncommonly associated with mental and physical excitement, it is reasonable to assume that in this case the hemorrhage was due to separation of thrombi in the uterine sinuses, induced directly by such a cause. When the patient was seen shortly after the occurrence of the hemorrhage, she had already passed rapidly from faintness, restlessness, and air hunger to cyanosis, convulsion, unconsciousness, and loss of wrist-pulse; respiration had ceased, and the patient was apparently dead. She was lying in a pool of blood, and the uterus extended into the epigastrium, and was full of blood-clots. Massage at this time failed to evoke the slightest response. The uterus was immediately forcibly squeezed and emptied, and pressure then applied through the flaccid and attenuated organ on to the aorta, the feeble pulsation of which showed that life was not yet extinct. With the patient in this moribund condition, any method of stimulating the uterus would have been utterly futile. Animation had first of all to be restored, and only prompt arrest of all bleeding and prompt and powerful treatment of shock and collapse could accomplish this. Hemorrage could not have been arrested by endeavouring to rouse the uterine forces. Contraction and retraction, the securing of which we look on as the final end to be obtained for the permanent arrest of hemorrhage, were completely absent. These forces had first to be restored before they could take part in the arrest of the hemorrhage, and their restoration had to be complete if they were to be relied on for permanently arresting the hemorrhage. For the restoration of these forces, it was first of all necessary for all bleeding to cease. Clotting of the blood in the uterine vessels is the only means of arresting hemorrhage when contraction and retraction are absent. It was necessary to arrest bleeding immediately. The methods of inducing clotting, as advocated in text-books, aim at applying pressure to the bleeding part. The use of perchloride of iron injection is regarded as dangerous and uncertain, and is obsolete. Pressure on the placenta site by plugging the utero-vaginal canal would have been useless here, as its success depends on a certain amount of remaining energy in the uterus ; and, again, it could not have been carried out completely before the patient died. Injection of hot water would also have been useless. Compression of the uterus is the method recommended for arresting hemorrhage when the uterine forces are completely exhausted. What were the chances of success of this method in this case at the time when compression of the aorta was adopted ? There were several features present which rendered compression of the uterus an uncertain method for rescuing the patient.

1. Prompt arrest of hemorrhage was imperative. To have introduced the unsterilised hand into the vagina and apply pressure to the uterus, even although the hand was not introduced into the uterus, would have been to incur great risk of sepsis. The patient certainly must have died long before the aseptic ritual was complete.

2. The bleeding area was situated on the lower portion of the lower uterine segment. Compression of the body of the uterus would not have ensured perfect pressure over the whole of the bleeding area for this reason, and for another which will be mentioned later. Compression of the uterus by pushing the cervix forward with one hand and anteflexing the body with the other, rarely finally arrests hemorrhage, and, as will be shown, did not admit of satisfactory application in this case. Compression with one hand inside the uterus on the placenta site and the other on the abdominal wall, is a very uncertain way of arresting hemorrhage when the uterine forces are entirely absent. The risk of sepsis being much greater than with any other method of compression, and owing to other circumstances present in this case, it could not be employed for arresting hemorrhage even temporarily.

3. The uterus extended into the epigastrium. It would have been perfectly impossible to compress such an unwieldy organ satisfactorily.

4. Notwithstanding prompt arrest of hemorrhage, contraction of the uterus did not reappear until after an hour's compression of the aorta, and it was not until some considerable time after this that contraction remained satisfactory. Owing to the desperate condition of the patient during the greater part of five hours, it was necessary to prevent any further loss of blood during this period. Compression of the uterus by any method cannot be kept up continuously for any length of time, and there is no certainty that loss of blood will not occur during the intervals of changing compressors.

5. Shock and collapse were extreme, and demanded prompt and powerful measures of treatment. Compression of the uterus alone would not have treated shock and collapse promptly and effectually enough in this case, and the life of the patient was at too low an ebb to have responded to such auxiliary measures as hypodermic administrations of strychnine, ether, &c.

Compression of the aorta was the only method that offered any chances of success in the treatment of the secondary post-partum hemorrhage in this case. The advantages of the method were well shown in this case. It could be applied immediately; its satisfactory application was quite unaffected by the position of the placental site, or by the dimensions of the uterus ; it could remain continuously in action for many hours, pressure being maintained longer by one individual than can be in compression of the uterus, and the changing of compressors not being accompanied by loss of blood ; it treated shock promptly and much more powerfully than compression of the uterus would have done.

Twelve hours after the moribund condition of the patient, the pulse had dropped to 100, and was strong and fairly full, all signs of collapse had disappeared, the uterus was firmly contracted, and no bleeding whatever had occurred since its arrest at the beginning of this period. Death occurred from heart-failure due directly to sudden and violent exertion about fourteen hours after the cessation of bleeding. At the time of its occurrence no further bleeding took place, the uterus being firmly contracted, and remaining so.

The fact also that posture, bandaging of the extremities, and saline transfusion formed a part of the treatment of the case does not affect the chief points at issue. Although these measures were employed, and were of unquestionable value, compression of the aorta formed the most important part of the treatment ; without it nothing would have been accomplished. It might be argued that it would have been a better plan to douche out the uterus with hot water while the hemorrhage was arrested by compressing the aorta. The effect of hot water on severe hemorrhage cannot always be relied on, and in this case it would have been unwise to employ a measure which is not always certain.

**The objections to compression of the aorta.** The chief objection urged against its use is its alleged detrimental interference with the functions of the uterus by curtailing its blood supply.[1] Although the blood-supply of the uterus by the uterine arteries is prohibited when the aorta is occluded, on anatomical grounds, quite enough blood should reach the organ by the ovarian arteries to enable it to contract and retract to the extent necessary at least for the *separation* of the placenta, and for the complete arrest of hemorrhage. The portions of the uterus chiefly affected when

[1] Fitzgerald, *Practitioner*, 1906, lxxvii. 652.

occlusion of the aorta takes place are the cervix, the lower uterine segment, and the lower portion of the upper uterine segment. The fundus receives a large part of its blood-supply from the ovarian arteries[1]. Free anastomosis exists between the branches of the ovarian and uterine arteries, especially in the upper uterine segment. When occlusion of the aorta takes place, the supply of blood is greatest in that portion of the uterus in which muscularity is greatest. From an anatomical point of view, therefore, a fair amount of blood should find its way to that part of the uterus which is most concerned in contraction and retraction. The question is whether *actually* a sufficient amount of blood is supplied to the uterus when the aorta is occluded to allow of the contraction and retraction necessary for the expulsion and separation of the placenta, and for the arrest of hemorrhage. This case supplies abundant clinical evidence on the last two points. When compression of the aorta took place before delivery of the placenta, during its application the uterus was observed to contract. At the end of half an hour the placenta still remained undelivered. The uterus was then grasped with one hand and pressure applied on the fundus, and in the direction of the sacrum. This proceeding resulted in immediately effecting complete extrusion of the placenta. The pressure exerted was not unusually forcible, being about the same as is required when the placenta has separated and is merely lying in the lower uterine segment. The question of the placenta not having separated did not suggest itself, owing to the ease with which it was expressed. The reason for introducing the hand into the uterus after delivery of the placenta was because the lower portion of the placenta—the early detached portion—was in such a ragged condition. As has been mentioned, there were no placental remains found when the uterus was explored, and this fact supports the conclusion that complete separation had occurred when expression took place. That the application of compression of the aorta before delivery of the placenta, and in the treatment of the secondary post-partum hemorrhage, did not interfere with the contraction and retraction necessary for arresting hemorrhage, is clearly proved by the record

[1] Jellet, *Manual of Midwifery*, 1905, p. 47.

of events. When compression was used before delivery of the placenta, no hemorrhage followed the release of compression, and an interval of eight hours' freedom from hemorrhage ensued, and the cause of the hemorrhage at the end of this period was quite unconnected with compression of the aorta. When compression was employed in the treatment of the secondary post-partum hemorrhage, contraction and retraction were completely absent. After an hour's compression, and during its application, the uterus hardened, became smaller, and massage, which previous to compression of the aorta failed to produce any effect, soon succeeded in bringing about firm contraction. No bleeding followed the release of compression. Further, the contraction and retraction resulting remained perfectly adequate up to the time of the patient's death, from heart-failure, eight hours after the discontinuance of compression. These forces indeed must have been unusually adequate, for in spite of the patient jumping out of bed, the loss of less than a tablespoonful of blood followed the incident. Even after death the uterus was still firmly contracted.

I think, therefore, that conclusive evidence was furnished that compression of the aorta did not affect injuriously the contractile and retractile functions of the uterus in their power for accomplishing separation of the placenta, and for arresting hemorrhage, either when its application took place before or after delivery of the placenta. The evidence derived from this case on these points is very valuable. The fact that the placenta separated naturally, and that contraction and retraction, which were previously completely absent, reappeared, and in spite of five hours' occlusion of the aorta, remained permanently adequate, is sufficient proof that compression of the aorta I think did not interfere with the power of the uterus for affecting separation of the placenta and for arresting hemorrhage.

The irksomeness of the method is another objection that has been brought against compression of the aorta. The maintenance of adequate pressure is not as irksome as would appear to those who have not employed compression of the aorta. It is certainly very much easier to keep up effectual pressure on the aorta than it is to compress the uterus bi-manually for even a short time. Although it was necessary in this case to keep up pressure for five hours, the circumstances were exceptional, one hour's compression being an outside limit in most cases, according to those who have used the method frequently.[1] An intelligent nurse can easily apply pressure, and as it is easy to prevent loss of blood when changing compressors—the new-comer placing the fist above or below that of the compressor before the latter releases pressure—no disadvantage attaches to changing compressors as often as may be necessary. The danger of reactionary hemorrhage and of syncope following the discontinuance of compression are avoided by withdrawing pressure gradually. With regard to the other objections brought forward, these are not of great importance, being easily overcome if compression is properly employed. These objections and the means of overcoming them were mentioned in my former paper.

In conclusion, I may say that I have used compression of the aorta on another occasion since in consultation, and this further experience has served to strengthen the conclusions arrived at in this case. The method as the primary part of the treatment of hemorrhage between the second and third stages of labour, due to partial separation of the placenta, possesses great advantages over immediate manual separation of the placenta, in those cases where serious exhaustion has been produced from tedious labour or loss of blood. If, after waiting half an hour, the placenta is not expelled naturally no harm is done, hemorrage being arrested; on the contrary, rest is given the tired uterus. Pressure on the uterus can then be tried, and if this fails to expel the placenta, or if the expressed placenta is incomplete, the uterus can then be emptied by introducing the hand, compression of the aorta continuing until the organ is completely emptied, and contraction and retraction complete.

[1] Stanmore Bishop, *Practitioner*, 1906, lxxvii.

